# From protection to rejection: the role of parental protection motivation in pre-prepared food consumption

**DOI:** 10.3389/fpsyg.2026.1928184

**Published:** 2026-10-14

**Authors:** Shaofeng Yuan, Weike Wu, Yidan Ma

**Affiliations:** 1 Faculty of Economics, Business School, Liaoning University, Shenyang, China; 2 Faculty of Economics, Asia-Australia Business College, Liaoning University, Shenyang, China

**Keywords:** parental protection motivation, pre-prepared food, perceived risk, health consciousness, information sensitivity

## Abstract

**Background:**

Pre-prepared foods are increasingly popular because of their convenience but have also raised concerns about food safety and nutritional risks. Drawing on the evolutionary needs perspective and protection motivation theory, this study examines the association between parental protection motivation and consumers’ purchase intention toward pre-prepared foods, as well as the mediating role of perceived risk and the moderating roles of health consciousness and information sensitivity in the mediation process.

**Methods:**

A cross-sectional online survey was conducted with 985 Chinese consumers. Confirmatory factor analysis in AMOS 28.0 assessed the measurement model, while PROCESS Model 4 and two Model 7 analyses in SPSS 30.0, using 5,000 bootstrap resamples, tested mediation and moderated mediation, respectively.

**Results:**

Parental protection motivation was negatively associated with purchase intention toward pre-prepared foods (*β* = −0.173, *p* < 0.001). Perceived risk significantly mediated this relationship [indirect effect = −0.280, 95% CI (−0.372, −0.196)]. The indices of moderated mediation were significant for both health consciousness [index = −0.185, 95% CI (−0.282, −0.112)] and information sensitivity [index = 0.066, 95% CI (0.016, 0.123)]. The mediating effect was significant at high but not low levels of health consciousness, whereas it was significant at low but not high levels of information sensitivity.

**Conclusion:**

Parental protection motivation was associated with lower purchase intention through perceived risk, and this mediating effect was stronger at higher levels of health consciousness but weaker at higher levels of information sensitivity. These findings deepen the protection-oriented explanation of parenting-related motives in consumer behavior and complement research on pre-prepared food consumption by directing attention beyond proximate explanations toward an ultimate-level motivational antecedent.

## 1 Introduction

The widespread adoption of industrial food production has accelerated the integration of pre-prepared foods into online delivery platforms, chain restaurants, and households. Their standardization, cost-effectiveness, and convenience are transforming food consumption (Wong et al., 2025). Pre-prepared food refers to products that undergo preliminary processing and packaging and require only heating or no further preparation before consumption (He et al., 2026). According to Fortune Business Insights (2026), the global pre-prepared food market was valued at $190.71 billion in 2025 and is projected to reach $326.50 billion by 2034, indicating significant commercial potential. However, rapid market growth has raised public concerns about food safety, nutritional quality, and additive use, which may threaten the industry’s sustainable development. Recent debates between Chinese influencer Luo Yonghao and the restaurant chain Xibei have sparked widespread discussion about the safety, quality, and reliability of these products (China Daily, 2025).

The tension between convenience and health protection is particularly salient in children’s diets, where parental protective instincts often lead to resistance against pre-prepared foods. Public criticism grew after Luo Yonghao revealed that Xibei’s children’s meals contained frozen broccoli with a shelf life of up to 24 months. Similarly, in September 2023, the introduction of pre-packaged meals in some primary and secondary schools in Jiangxi Province faced strong opposition, with over 80% of parents disapproving in a survey of 1.09 million participants (Sina Weibo^1^). As the primary custodians of children’s nutritional well-being, parents assess pre-prepared foods through the lens of their potential effects on their children’s health. Such assessments embody the concept of parental protection motivation, which is characterized as the impetus to alter behavior in order to safeguard one’s offspring (Buckels et al., 2015). Consequently, when pre-prepared foods are appraised with regard to children’s health and safety, consumer evaluations may transition from a focus on convenience to one centered on protection-driven risk assessment. While these cases illustrate the practical relevance of parental protection in household food decisions, current literature has yet to examine whether this protection-oriented motivation is associated with consumers’ risk perceptions and their purchase intentions toward pre-prepared foods.

Prior research has provided valuable insights into the factors influencing consumers’ acceptance and consumption of pre-prepared foods. Existing studies have examined consumers’ purchase intention toward pre-prepared food, highlighting the importance of intrinsic product characteristics such as price, taste, and packaging (Liu et al., 2025; Xiong et al., 2023; Xu and Sun, 2026), as well as psychological factors including perceived value, trust, and attitude (Liu et al., 2025; Wong et al., 2025; Zhang et al., 2024). Additionally, the negative impact of perceived risk on purchase intention has been well established in the literature (He et al., 2026; Xiong et al., 2023). The evolutionary needs perspective distinguishes between proximate explanations, which focus on the immediate psychological processes underlying behavior, and ultimate explanations, which address the recurrent adaptive problems that these processes have evolved to solve, including survival, reproduction, and offspring care (Confer et al., 2010; Griskevicius and Kenrick, 2013). Within this perspective, the product characteristics and cognitive assessments investigated in current research on pre-prepared foods are predominantly situated at the proximate level.

Meanwhile, consumer research on broad parenting motives has increasingly demonstrated that the fundamental motivation to care for children influences product selection, temporal orientations, risk preferences, and consumption decisions related to children (Li et al., 2019; Liang et al., 2023; Roy et al., 2026). These broad motives encompass a wide range of child-centered concerns and goals, such as investing time and resources in children’s education and long-term development. Within the framework of the parental care motivational system, Hofer et al. (2018) identify two conceptually distinct dimensions: nurturance and protection. Nurturance pertains to the inclination to perceive infants as emotionally rewarding and to respond with tenderness and supportive caregiving, whereas protection involves the drive to safeguard children from harm, closely associated with threat detection, harm prevention, and heightened risk vigilance. Given that pre-prepared foods frequently elicit concerns regarding food safety, nutritional quality, and transparency of information (He et al., 2026; Wong et al., 2025), the protective component of parental caregiving is especially pertinent to consumers’ assessments of these products.

Taken together, these two research streams reveal three related gaps. First, research on pre-prepared food consumption has focused primarily on proximate product attributes and cognitive evaluations, while the potential relevance of evolutionarily based motives has been inadequately explored. Second, consumer research on parenting-related motives has yet to clarify the relationship between the protective component of parental caregiving and consumers’ evaluations of pre-prepared foods. Third, there has been insufficient investigation into the cognitive mechanisms and boundary conditions that govern this relationship. These gaps are particularly pertinent in a consumption context where the convenience offered by pre-prepared foods intersects with concerns about children’s health and safety.

An evolutionary needs perspective provides a useful basis for addressing these gaps. The evolutionary needs perspective posits that human decision-making is influenced not only by immediate assessments but also by fundamental motives that have evolved to address recurrent adaptive challenges, including self-protection, disease avoidance, social affiliation, and kin care (Confer et al., 2010; Griskevicius and Kenrick, 2013). The parental protection (kin care) system evolved to ensure offspring survival and well-being, guiding parents to prioritize safety, health, and nutrition. Applying this framework clarifies why parents may resist or selectively choose certain convenience or cost-effective pre-prepared foods (Liang et al., 2023) thereby providing a more nuanced understanding of contemporary consumer decision-making processes. Additionally, protection motivation theory posits that protective behaviors are influenced by threat appraisal, offering a psychological mechanism through which perceived risk mediates the relationship between parental protection motives and purchase intentions (Floyd et al., 2000; Rogers, 1975). Thus, the evolutionary needs perspective explains the motivational origin of parental protection concerns, whereas protection motivation theory explains how such concerns may be translated into risk-based consumer responses.

Accordingly, the objective of this study is to examine the relationship between parental protection motivation and consumers’ purchase intention toward pre-prepared foods, the mediating role of perceived risk, and the boundary conditions of this mediation process. Specifically, the study addresses the following research questions:

*RQ1*: What is the relationship between parental protection motivation and consumers’ purchase intention toward pre-prepared foods?

*RQ2*: Does perceived risk mediate this relationship?

*RQ3*: Do health consciousness and information sensitivity moderate the mediating effect of perceived risk on the relationship between parental protection motivation and purchase intention toward pre-prepared foods?

## 2 Theoretical background

To situate the current study within the extant literature, previous research is organized into two streams: (1) parenting-related motivation and consumer behavior and (2) antecedents of pre-prepared food consumption. Within the first stream, broad parenting-related motivation refers to studies that examine parenting or caregiving goals without isolating their protective component, whereas parental protection motivation refers specifically to the motivation to shield children from potential harm or risk. Within the second stream, proximate factors refer to product characteristics and immediate psychological assessments influencing purchasing decisions, such as price, convenience, perceived value, trust, attitude, and perceived risk. Conversely, ultimate-level explanations focus on evolutionarily based motives, including those associated with survival, reproduction, and offspring protection.

Supplementary material summarizes representative studies that exemplify these two research streams and highlight their primary focal areas. These studies were chosen due to their direct investigation of key motivational constructs, consumption contexts, or boundary conditions relevant to the present research. The purpose of this selection is to contextualize the present study within the existing literature, rather than to offer a comprehensive literature review in these domains.

### 2.1 Evolutionary needs and parental protection motivation

The evolutionary needs framework posits that human psychological mechanisms have developed to address recurrent ancestral challenges related to survival and reproduction (Confer et al., 2010; Griskevicius and Kenrick, 2013). Fundamental evolutionary needs include self-protection, disease avoidance, social affiliation, status acquisition, mate acquisition, mate retention, and kin care.

Parental protection motivation, which emerges from the kin-care system, refers to a motivational tendency that directs individuals to protect children from potential harm (Buckels et al., 2015). This system’s adaptive purpose is to promote the survival, health, and development of offspring. Given that offspring survival is directly linked to reproductive success, humans have evolved an increased sensitivity to dangers that may compromise children’s safety, health, and nutritional status (Taheran et al., 2024). Although this motivational system may be more chronically activated among parents, it is not limited to individuals who currently have children. Prior research indicates that the parental care motivational system can also be activated among nonparents, eliciting protective and nurturing behaviors (Buckels et al., 2015; Schaller, 2018).

Broad parenting motivation provides a general framework for understanding the influence of caregiving motivation on parental behavior. It encompasses a wide range of child-oriented concerns and goals, such as investing time and resources in children’s education and long-term development. Existing research focuses primarily on the broader concept of parenting motivation, demonstrating its effects on consumer decisions related to safety assurance (Roy et al., 2026), long-term developmental investment (Chen et al., 2024; Li et al., 2019), and emotional bonding (Hofer et al., 2018). For example, consumers with strong parenting motivation tend to avoid risk under time pressure by choosing familiar and efficient products (Liang et al., 2023).

Broad parenting motivation and parental protection motivation are interconnected yet conceptually distinct. Broad parenting motivation refers to the overall drive to nurture and care for children across various parental objectives and decision-making processes. In contrast, parental protection motivation represents the protective component of the parental care motivational system, emphasizing the identification of threats, prevention of harm, and safeguarding of offspring (Hofer et al., 2018). While pre-prepared foods provide convenience, their industrial processing often raises issues related to food safety, ingredient transparency, and nutritional quality (He et al., 2026). From an evolutionary needs perspective, stronger parental protection motivation should be associated with heightened vigilance toward such concerns when children’s health is salient. Taken together, existing research demonstrates that broad parenting-related motives are relevant to consumer decision-making, but it remains unclear whether the protection-specific component is associated with consumers’ perceived risk and purchase intention toward pre-prepared foods. Examining these relationships may therefore clarify how an evolutionarily based parental motive is reflected in family-centered food choices that balance convenience with health-related considerations.

### 2.2 Antecedents of pre-prepared food consumption

Prior research has identified a range of factors that influence consumers’ purchase intention toward pre-prepared foods. The extant literature predominantly focuses on intrinsic product characteristics, including price, taste, packaging, and convenience (Liu et al., 2025; Xiong et al., 2023; Xu and Sun, 2026), as well as psychological factors including perceived value, trust, and attitude (Liu et al., 2025; Wong et al., 2025; Zhang et al., 2024). Additionally, perceived risk has been demonstrated to negatively affect purchase intentions, particularly in relation to concerns about food safety, nutritional quality, and additive use (He et al., 2026; Xiong et al., 2023).

Moreover, research on food parenting and healthy eating behaviors suggests that parental motivations and practices significantly shape children’s dietary patterns. Strategies such as restrictive feeding, control over food availability, and parental modeling have been shown to substantially influence children’s consumption of unhealthy or energy-dense foods (Yee et al., 2017). Further studies indicate that parental caregiving motives increase sensitivity to potential threats to children’s well-being (Fessler et al., 2014; Hofer et al., 2018), which may, in turn, promote heightened vigilance regarding food safety and nutritional appropriateness (Maubach et al., 2009; Roy et al., 2026). Studies on ready-to-eat and prepackaged foods also highlight the impact of factors such as time constraints, cooking self-efficacy, meal planning, and outcome expectations on purchasing behavior (Horning et al., 2017; Johnson et al., 2019).

Despite these insights, several limitations remain. First, most studies focus on proximate antecedents, while neglecting ultimate motivations grounded in evolutionary psychology. Second, although prior research has indirectly implied that parental motivations affect food choices (Johnson et al., 2019; Liang et al., 2023), the specific role of parental protection motivation as an evolutionary driver in pre-prepared food consumption remains underexplored. Third, existing investigations tend to focus on general food parenting practices or healthy eating behaviors rather than specifically addressing pre-prepared food consumption. Finally, limited attention has been paid to the cognitive mechanisms underlying these relationships, particularly the potential mediating role of perceived risk in the relationship between parental protection motivation and purchase intention. These gaps highlight the need to integrate evolutionary motivations into research on consumer decision-making regarding pre-prepared foods.

## 3 Research framework and hypothesis development

To address the gaps identified in previous research, this study develops a conceptual framework (Figure 1) to examine: (a) the relationship between parental protection motivation and consumers’ purchase intention toward pre-prepared foods; (b) whether perceived risk mediates this relationship; and (c) whether health consciousness and information sensitivity moderate this process.

**Figure 1:**
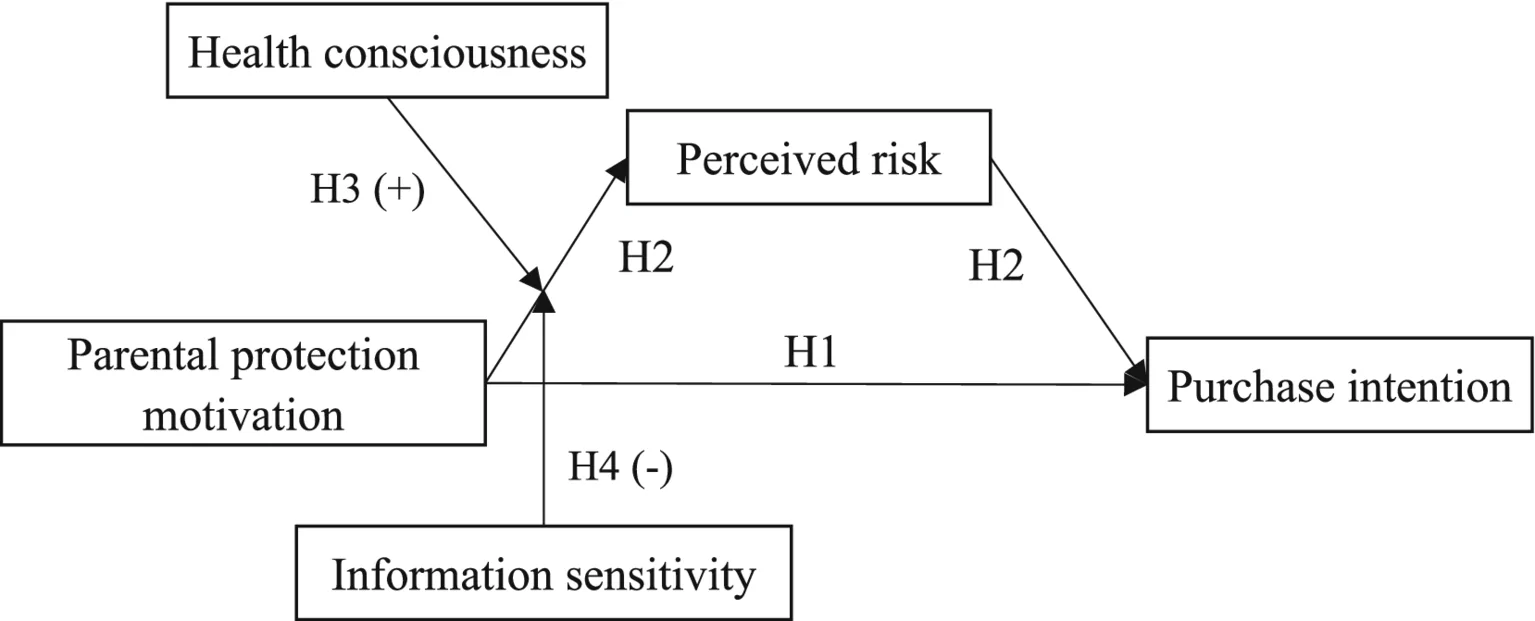
Conceptual framework. Source: Authors’ own work.

### 3.1 Parental protection motivation and purchase intention toward pre-prepared foods

Pre-prepared foods, as a category of industrially processed products, frequently exhibit limited transparency concerning their ingredients and manufacturing methods, which may conflict with evolutionary tendencies toward disease avoidance and offspring protection. From the evolutionary needs perspective, when food choices pertain to the well-being of children, threat-detection mechanisms are activated, resulting in increased caution toward unfamiliar or extensively processed foods (Kenrick et al., 2010). Moreover, the parental care motivational system can be engaged not only in individuals who are parents but also in nonparents (Buckels et al., 2015; Schaller, 2018). Consequently, parental protection motivation may influence food evaluations among a broad range of consumers, rather than being limited solely to those who currently have children.

Parental protection motivation should also influence how consumers translate child-protection concerns into marketplace choices. Research on parenting motivation shows that the salience of parenting goals affects intertemporal preferences and diminishes variety-seeking behavior, suggesting that such motivations can systematically modify consumer decision-making strategies (Li et al., 2019; Liang et al., 2023). More specifically, evidence pertaining to protective motives indicates that heightened parental-role salience leads to increased risk aversion (Eibach and Mock, 2011), while a focus on safety prompts parents to favor best-selling products that convey reassuring signals of popularity and approval (Roy et al., 2026). Collectively, these findings imply that parenting-related motivations tend to steer consumers toward choices that provide greater reassurance and away from options that are less aligned with protective concerns. In the context of pre-prepared food consumption, it follows that stronger parental protection motivation should correspond with a lower willingness to purchase such products. Formally, the following hypothesis is proposed:

*Hypothesis 1*: Parental protection motivation is negatively associated with consumers’ purchase intention toward pre-prepared foods.

### 3.2 The mediating role of perceived risk

Protection motivation theory (Rogers, 1975) explains how threat appraisal generates protective behavioral intentions. Rooted in the fundamental drive for safety and harm avoidance, this theory posits that individuals assess potential threats and subsequently adopt behaviors aimed at mitigating perceived risks (Griskevicius and Kenrick, 2013). Threat appraisal involves judgments of perceived severity and vulnerability, providing a theoretical basis for understanding how concerns about potential harm are related to protective responses (Floyd et al., 2000). In the present consumption context, these concerns are relevant to consumers’ assessments of the potential negative consequences of purchasing pre-prepared foods. Perceived risk refers to consumers’ subjective feelings of uncertainty and anticipated negative outcomes during the purchasing process (Ge, 2022; Stone and Grønhaug, 1993). Perceived risk is therefore adopted as the mediating construct because it captures the overall purchase-related evaluation central to the research question. Perceived risk applies the theory’s threat-evaluation logic to consumers’ judgments of the potential negative consequences of purchasing pre-prepared foods.

Individuals with stronger parental protection motivation may be more vigilant toward potential threats to children’s health and safety (Fessler et al., 2014; Hofer et al., 2018). In the context of pre-prepared foods, factors such as industrial processing, limited transparency regarding ingredients, and ambiguous nutritional quality may amplify perceived risk (He et al., 2026; Wong et al., 2025). Stronger parental protection motivation may therefore be associated with greater uncertainty and stronger expectations of potential negative consequences, thereby resulting in higher perceived risk.

Higher perceived risk, in turn, is generally associated with risk-avoidance responses and lower purchase intention. Previous research has consistently shown that increased perceived risk negatively impacts consumers’ willingness to buy food products (Castagna et al., 2021). Meta-analytic evidence further confirms a significant negative relationship between perceived risk and purchase intention (Pelaez et al., 2019). Thus, although pre-prepared foods provide convenience and efficiency, higher perceived risk may prompt consumers to engage in risk-avoidance strategies, thereby decreasing their purchase intention.

Taken together, stronger parental protection motivation is expected to be associated with greater perceived risk, which is subsequently associated with lower purchase intention toward pre-prepared foods. Consequently, perceived risk may serve as a mediating factor explaining the negative relationship between parental protection motivation and purchase intention. Therefore, the following hypothesis is proposed:

*Hypothesis 2*: Perceived risk mediates the relationship between parental protection motivation and purchase intention toward pre-prepared foods, such that stronger parental protection motivation is associated with greater perceived risk and, in turn, lower purchase intention.

### 3.3 The moderated mediation effect of health consciousness

Protection motivation theory further suggests that threat appraisal varies according to individual cognitive characteristics and information-processing tendencies (Milne et al., 2000). In this study, health consciousness and information sensitivity are proposed as important boundary conditions of the relationship between parental protection motivation and perceived risk.

Health consciousness refers to the extent to which individuals are concerned about and attentive to health issues (Michaelidou and Hassan, 2008). Prior research indicates that highly health-conscious consumers possess greater health knowledge, scrutinize food information more carefully, and adopt more health-oriented lifestyles (Klerck and Sweeney, 2007; Siegrist et al., 2022). Health consciousness also influences product evaluations and food purchasing decisions (Nagaraj, 2021).

When consumers with high health consciousness are also strongly motivated to protect their offspring, they are more likely to apply stringent health standards to food products and focus on potential risk cues, such as additives and nutritional quality (Mäkelä et al., 2023; Shimul et al., 2021). As a result, health consciousness may strengthen the positive relationship between parental protection motivation and perceived risk. Given that greater perceived risk is associated with lower purchase intention in food consumption contexts (Ge, 2022), the negative indirect relationship between parental protection motivation and purchase intention through perceived risk should be stronger among consumers with higher health consciousness. Accordingly, the following hypothesis is proposed:

*Hypothesis 3*: Health consciousness moderates the indirect effect of parental protection motivation on purchase intention through perceived risk, such that this negative indirect effect is stronger at higher than at lower levels of health consciousness.

### 3.4 The moderated mediation effect of information sensitivity

Sensitivity refers to an individual’s tendency to respond strongly to external stimuli and assign importance to them (Pluess, 2015). In consumer research, this concept is often operationalized as consumers’ attention to specific product attributes or informational cues (Bansal et al., 2010; Gouthier et al., 2022; Vogrincic-Haselbacher et al., 2021). Building on this perspective, the present study defines information sensitivity as the degree to which consumers prioritize objective product information, such as ingredient lists, processing methods, and food-origin information.

According to the elaboration likelihood model, consumers process information through different routes depending on their motivation and cognitive engagement (Shahab et al., 2021). Consumers with high information sensitivity tend to rely on substantive information rather than peripheral cues when evaluating products. For example, Jin and Zhou (2014) found that consumers with strong food safety concerns actively seek traceability information to reduce uncertainty. Similarly, among consumers with stronger parental protection motivation, those with higher information sensitivity may place greater weight on available product-specific information when evaluating food safety. Such attention may make risk judgments more dependent on product-specific information and less closely tied to general protective concerns (Liu et al., 2022; Zhu et al., 2025).

In contrast, consumers with low information sensitivity pay limited attention to technical or safety-related information. When evaluating product safety, these consumers may rely more on general impressions or heuristics, allowing protection-related concerns to be more closely associated with perceived risk (Dhar and Gorlin, 2013). Information sensitivity is therefore expected to weaken the positive relationship between parental protection motivation and perceived risk. Given the negative relationship between perceived risk and purchase intention (Ge, 2022), the negative indirect relationship between parental protection motivation and purchase intention through perceived risk should be weaker among consumers with higher information sensitivity. Accordingly, the following hypothesis is proposed:

*Hypothesis 4*: Information sensitivity moderates the indirect effect of parental protection motivation on purchase intention through perceived risk, such that this negative indirect effect is weaker at higher than at lower levels of information sensitivity.

## 4 Methods

### 4.1 Participants and data collection

This study employed a cross-sectional online survey of Chinese consumers. Formal data collection was conducted from September 21 to September 27, 2025, following the controversy involving the Chinese influencer Luo Yonghao and the restaurant chain Xibei. Participants were recruited via Credamo.com, an open online research platform comprising a sample pool exceeding three million registered members from various provinces and regions across China. For paid surveys, the platform randomly distributes survey invitations among its registered panel members, providing access to consumers across different regions of China (Wang et al., 2025). Credamo has also been used in prior consumer behavior research conducted in China (e.g., Fu et al., 2024; Sun et al., 2023). All participants provided informed consent and received 2 RMB in compensation. The individual consumer served as the unit of analysis.

A total of 1,000 responses were collected. After excluding 15 invalid responses due to failed attention checks or excessively short completion times (less than 120 s), the final sample consisted of 985 participants (M_age_ = 31.52 years, SD = 8.29), of whom 64.5% were female.

No additional demographic screening criteria were specified for the present study. Whether participants had children and the possibility of future parenting were not used as eligibility criteria. The sample comprised adult consumers irrespective of whether they had children, based on evidence suggesting that the parental care motivational system can be activated in both parents and nonparents (Buckels et al., 2015; Schaller, 2018). These two variables were recorded and included as covariates in the primary analyses.

### 4.2 Measures

All constructs were measured using established scales adapted from prior studies. The linguistic and contextual appropriateness of these scales for Chinese participants was evaluated during the adaptation process. The original items were translated into Chinese and subsequently back-translated into English. Any inconsistencies identified between the original and back-translated versions were reviewed and resolved by two doctoral-level researchers familiar with consumer psychology and food-consumption research. Subsequently, the questionnaire underwent further discussion and refinement by a panel of seven experts, including two professors and five doctoral candidates specializing in consumer psychology. Finally, a pilot study was conducted with 30 Chinese consumers to evaluate the clarity, comprehensibility, and contextual relevance of the items, leading to minor revisions in wording based on participant feedback.

The five primary constructs were measured using 20 items, with each construct measured by at least three items. Except for the control variables, all measures used a seven-point scale (1 = strongly disagree, 7 = strongly agree). Specifically, purchase intention was measured using three items adapted from Dodds et al. (1991). Parental protection motivation was assessed using five items from Buckels et al. (2015). Perceived risk was measured with three items adapted from Stone and Grønhaug (1993) overall risk scale. Health consciousness was measured using four items adapted from Michaelidou and Hassan (2008), while information sensitivity was measured using five items adapted from Bansal et al. (2010). The complete measurement items are provided in Supplementary material.

Participants also reported demographic information, including gender (male = 1, female = 2), age, whether they have children (yes = 1, no = 0), the possibility of future parenting (0% = 0, 1–20% = 1, 21–40% = 2, 41–60% = 3, 61–80% = 4, 81–99% = 5, and 100% = 6), and awareness of the Luo Yonghao–Xibei controversy (yes = 1, no = 0). Gender, age, and whether they have children were included because demographic and household characteristics may be associated with food-safety risk perception (Nardi et al., 2020). The possibility of future parenting was included because parental care motivation among nonparents is associated with the desire to have children (Buckels et al., 2015). Controversy awareness was controlled for, given that exposure to food-safety information through social media may affect risk perception (Zhang et al., 2022).

### 4.3 Measurement assessment

Data were analyzed using SPSS 30.0 and AMOS 28.0. Reliability was assessed using Cronbach’s α, with values above 0.70 indicating acceptable internal consistency (Nunnally and Bernstein, 1994). Construct validity was evaluated through confirmatory factor analysis (CFA). Following Hu and Bentler (1999), model fit was considered acceptable when *χ*^2^/df < 5, comparative fit index (CFI) and Tucker–Lewis index (TLI) > 0.90, and root mean square error of approximation (RMSEA) < 0.08.

Convergent and discriminant validity were assessed according to Hair et al. (2012). Composite reliability (CR) values above 0.70 and average variance extracted (AVE) values above 0.50 indicated adequate convergent validity. Discriminant validity was evaluated using both the Fornell–Larcker criterion and the heterotrait–monotrait ratio of correlations (HTMT). It was supported when the square root of each construct’s AVE exceeded its correlations with the other constructs and when all HTMT values were below 0.85 (Henseler et al., 2015).

To assess common method bias, CFA was used to compare the fit of a single-factor model with that of the proposed five-factor model (Podsakoff et al., 2012). A substantially poorer fit for the single-factor model would indicate that common method bias was less likely to threaten the validity of the findings. As an additional diagnostic measure, full-collinearity variance inflation factors (VIFs) were computed for the five primary constructs utilizing their composite scores. Following Kock (2015), each construct was regressed on the remaining constructs, with values below 3.3 indicating that common method bias was unlikely to pose a serious concern.

### 4.4 Hypothesis testing methods

The hypotheses were tested using Hayes’ PROCESS macro (Hayes, 2022). Model 4 was used to examine the mediating role of perceived risk, whereas two Model 7 analyses were conducted to test the moderating roles of health consciousness and information sensitivity in the mediation process, respectively. The analyses employed 5,000 bootstrap resamples and bias-corrected 95% confidence intervals (CIs), with gender, age, whether participants had children, the possibility of future parenting, and controversy awareness entered simultaneously as covariates in all primary models.

Furthermore, consistent with previous research employing PROCESS Model 59 to investigate variations in mediation processes across dichotomous demographic groups (Tian et al., 2022), a supplementary conditional process analysis was conducted. This analysis examined whether the mediation process differed between participants with and without children, with gender, age, the possibility of future parenting, and controversy awareness included as covariates.

## 5 Results

### 5.1 Reliability and validity

Table 1 presents the reliability, convergent validity, and discriminant validity results. All Cronbach’s α values exceeded 0.80, indicating excellent reliability. The CFA results demonstrated good model fit (*χ*^2^ = 702.654, df = 160, *χ*^2^/df = 4.392, CFI = 0.947, TLI = 0.937, RMSEA = 0.059), supporting construct validity.

**Table 1:** Convergent and discriminant validity.

Variables	1	2	3	4	5	CR	AVE	α	VIF
1. Purchase intention	**0.903**					0.930	0.816	0.929	1.764
2. Parental protection motivation	−0.165^**^	**0.679**				0.810	0.461	0.807	1.208
3. Perceived risk	−0.633^**^	0.258^**^	**0.859**			0.894	0.738	0.894	1.764
4. Health consciousness	−0.261^**^	0.224^**^	0.185^**^	**0.720**		0.810	0.518	0.810	1.178
5. Information sensitivity	−0.337^**^	0.375^**^	0.341^**^	0.341^**^	**0.724**	0.845	0.524	0.841	1.372

CR, composite reliability; AVE, average variance extracted; α, Cronbach’s α, VIF = full-collinearity variance inflation factor, bold diagonals are the square roots of AVEs, ^*^ and ^**^ represent *p* < 0.05 and *p* < 0.01, respectively.

Source: Authors’ own work.

Convergent validity was supported because all CR values exceeded 0.70, and all AVE values exceeded 0.50 except for parental protection motivation (AVE = 0.461). Although its AVE was slightly below 0.50, the CR of parental protection motivation was 0.810, well above 0.60. Following Fornell and Larcker (1981), convergent validity may still be considered acceptable.

Discriminant validity was also supported, as the square roots of AVEs exceeded the inter-construct correlations, and the HTMT ratios ranged from 0.187 to 0.694, with all values below 0.85 (see Supplementary material).

### 5.2 Common method bias

The single-factor model demonstrated poor fit (*χ*^2^ = 5630.377, df = 170, *χ*^2^/df = 33.120, CFI = 0.464, TLI = 0.401, RMSEA = 0.181), with all indices falling outside recommended thresholds (Hu and Bentler, 1999). The same five-factor measurement model reported in Section 5.1 served as the comparison model and showed substantially better fit. As reported in Table 1, the full-collinearity VIFs ranged from 1.178 to 1.764 and were all below the recommended threshold of 3.3. Taken together, the model-comparison and full-collinearity results suggest that common method bias was unlikely to pose a serious concern.

### 5.3 Hypothesis testing

#### 5.3.1 Main effect test

The regression model was significant (*F* = 8.060, *p* < 0.001). Parental protection motivation was significantly and negatively associated with purchase intention (*β* = −0.173, *p* < 0.001). Therefore, Hypothesis 1 was supported.

#### 5.3.2 Mediating effect of perceived risk

As shown in Table 2, parental protection motivation had a significant indirect effect on purchase intention through perceived risk (IE = −0.280, 95% CI = [−0.372, −0.196]). In contrast, the direct effect was not significant because the 95% CI included zero (effect = −0.003, 95% CI = [−0.090, 0.084]). These findings indicate that perceived risk mediates the relationship between parental protection motivation and purchase intention, supporting Hypothesis 2.

**Table 2:** The mediating effect of perceived risk.

Relationship	Coefficient	SE	*t*	95% CI
Total effect	−0.282	0.055	−5.176^**^	[−0.390, −0.175]
Direct effect	−0.003	0.044	−0.060	[−0.090, 0.084]
PPM → perceived risk	0.380	0.046	8.289^**^	[0.290, 0.470]

	Effect	SE	95% CI
Indirect effect	−0.280	0.045	[−0.372, −0.196]

PPM, Parental protection motivation. ^*^ and ^**^ represent *p* < 0.05 and *p* < 0.01, respectively.

Source: Authors’ own work.

A supplementary analysis found that whether participants had children did not significantly moderate the association between parental protection motivation and perceived risk (*β* = −0.058, *p* = 0.539), the association between perceived risk and purchase intention (*β* = −0.013, *p* = 0.834), or the direct association between parental protection motivation and purchase intention (*β* = 0.049, *p* = 0.595). The indirect effect through perceived risk was significant among participants without children (IE = −0.303, 95% CI [−0.430, −0.190]) and those with children (IE = −0.265, 95% CI [−0.390, −0.152]), whereas the difference between the two indirect effects was not significant (index = 0.037, 95% CI [−0.131, 0.204]).

#### 5.3.3 Moderated mediation: the role of health consciousness

As shown in Table 3, health consciousness significantly moderated the indirect effect of parental protection motivation on purchase intention through perceived risk (index = −0.185, 95% CI = [−0.282, −0.112]). Specifically, the indirect effect was significant at high levels of health consciousness (IE = −0.440, 95% CI = [−0.578, −0.323]), but not at low levels (IE = −0.096, 95% CI = [−0.206, 0.014]).

**Table 3:** The moderating effect of health consciousness.

Relationship	Coefficient	SE	*t*	95% CI
Direct effects				
PPM → perceived risk	0.363	0.046	7.921^**^	[0.273, 0.453]
Perceived risk → purchase intention	−0.737	0.030	−24.611^**^	[−0.796, −0.678]
PPM → purchase intention	−0.003	0.044	−0.060	[−0.090, 0.084]
Indirect effects				
Health consciousness → perceived risk	0.248	0.045	5.561^**^	[0.160, 0.336]
PPM × Health consciousness → perceived risk	0.251	0.043	5.808^**^	[0.166, 0.336]

Conditional indirect effects of PPM → purchase intention (via perceived risk), by health consciousness			
Health consciousness	IE	SE	95% CI
Low (M − 1SD)	−0.096	0.056	[−0.206, 0.014]
High (M + 1SD)	−0.440	0.064	[−0.578, −0.323]
Index of moderated mediation	−0.185	0.044	[−0.282, −0.112]

Source: Authors’ own work.

Figure 2 further illustrates the interaction effect. The positive association between parental protection motivation and perceived risk was stronger at high levels of health consciousness (effect = 0.597, 95% CI = [0.472, 0.722]) than at low levels (effect = 0.130, 95% CI = [0.026, 0.244]). These findings support Hypothesis 3.

**Figure 2:**
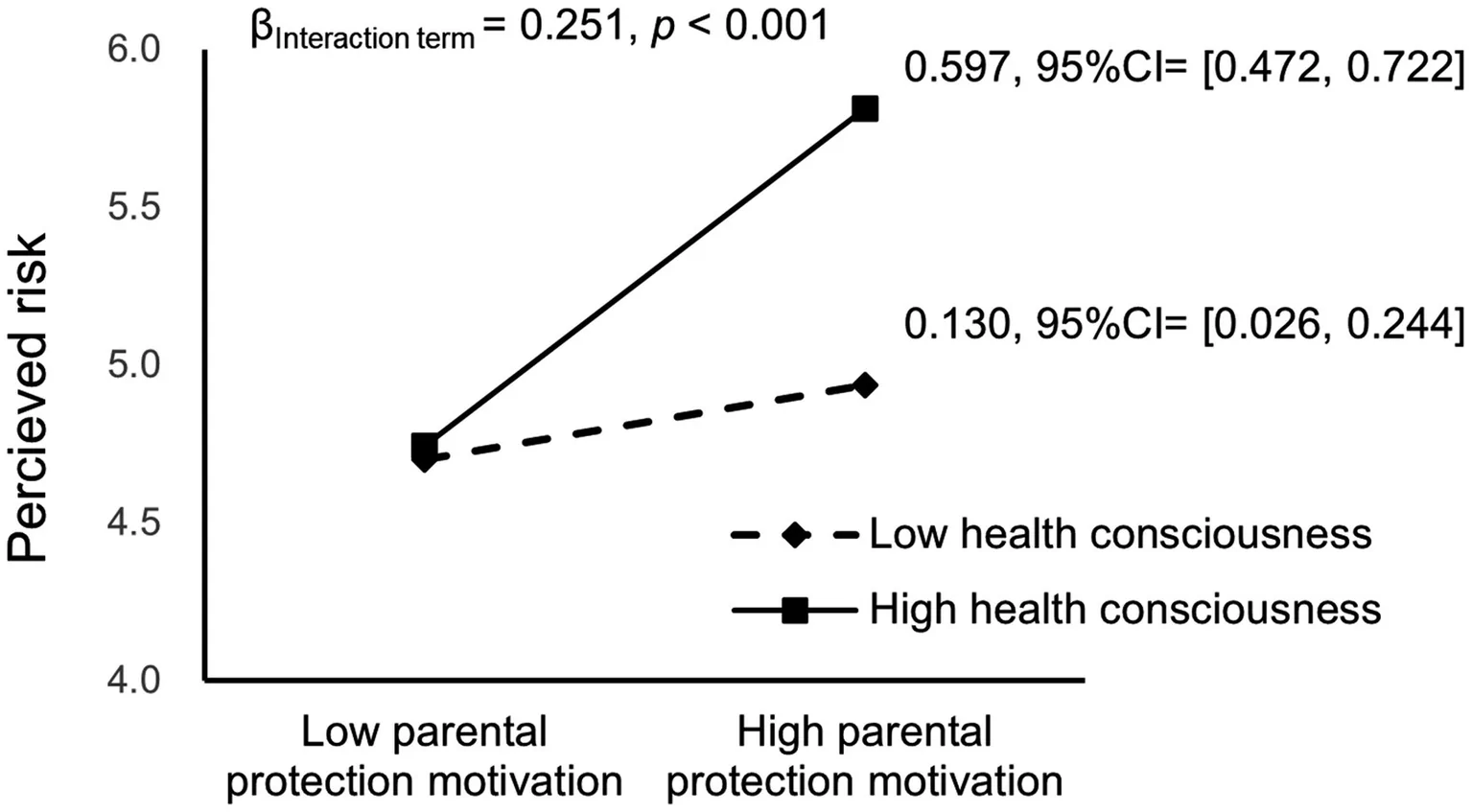
The moderating effect of health consciousness. Source: Authors’ own work.

#### 5.3.4 Moderated mediation: the role of information sensitivity

As shown in Table 4, information sensitivity significantly moderated the indirect effect of parental protection motivation on purchase intention through perceived risk (index = 0.066, 95% CI = [0.016, 0.123]). The indirect effect was significant at low levels of information sensitivity (effect = −0.207, 95% CI = [−0.291, −0.125]), but not at high levels (effect = −0.078, 95% CI = [−0.187, 0.026]).

**Table 4:** The moderating effect of information sensitivity.

Relationship	Coefficient	SE	*t*	95% CI
Direct effects				
PPM → perceived risk	0.194	0.049	3.963^**^	[0.098, 0.290]
Perceived risk → purchase intention	−0.737	0.030	−24.611^**^	[−0.796, −0.678]
PPM → purchase intention	−0.003	0.044	−0.060	[−0.090, 0.084]
Indirect effects				
Information sensitivity → perceived risk	0.332	0.046	7.294^**^	[0.243, 0.422]
PPM × Information sensitivity → perceived risk	−0.090	0.031	−2.853^**^	[−0.151, −0.028]

Conditional indirect effects of PPM → purchase intention (via perceived risk), by information sensitivity			
Information sensitivity	IE	SE	95% CI
Low (M − 1SD)	−0.207	0.043	[−0.291, −0.125]
High (M + 1SD)	−0.078	0.054	[−0.187, 0.026]
Index of moderated mediation	0.066	0.027	[0.016, 0.123]

Source: Authors’ own work.

Figure 3 illustrates the interaction effect. Parental protection motivation was positively associated with perceived risk at low levels of information sensitivity (effect = 0.281, 95% CI = [0.183, 0.379]), whereas the effect was nonsignificant at high levels (effect = 0.106, 95% CI = [−0.020, 0.233]). These findings support Hypothesis 4.

**Figure 3:**
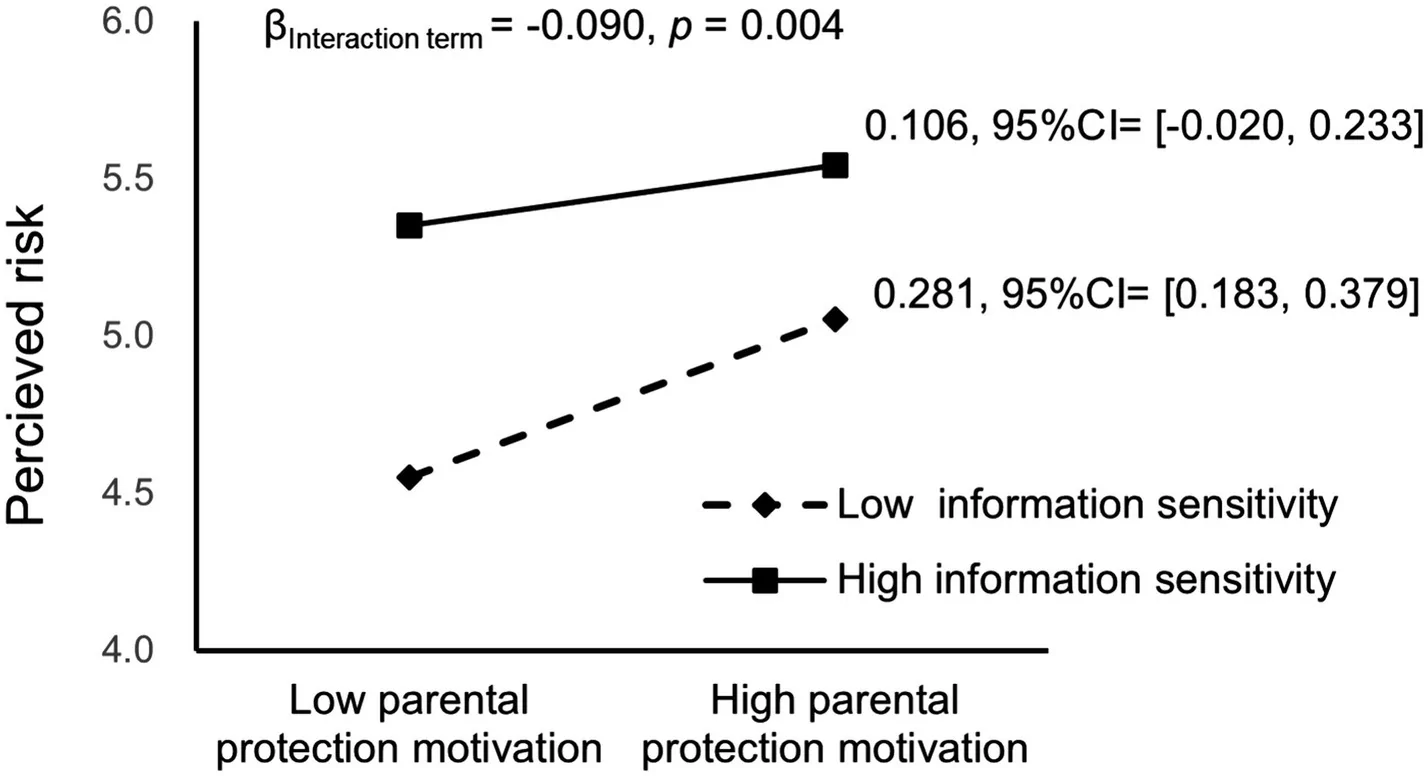
The moderating effect of information sensitivity. Source: Authors’ own work.

## 6 Discussion

This study examined the relationship between parental protection motivation, a fundamental evolutionary motive, and consumers’ purchase intention toward pre-prepared foods. Prior research on pre-prepared food consumption has primarily focused on proximate factors, such as price, taste, and product-related evaluations, while paying relatively limited attention to evolutionarily based motives. Although consumer research has examined broader parenting-related motives, the protection-oriented component of parental caregiving has received less attention in the context of food consumption. Addressing these gaps, this study investigated the mediating role of perceived risk and the moderated mediation effects of health consciousness and information sensitivity in the relationship between parental protection motivation and purchase intention toward pre-prepared foods.

First, the findings showed that parental protection motivation was negatively associated with purchase intention toward pre-prepared foods and that perceived risk mediated this relationship. These findings suggest that parenting-related motives may be relevant not only to caregiving, support, and resource investment, but also to defensive responses in consumer decision-making. In the context of pre-prepared foods, consumers’ risk judgments may reflect not only product attributes and external information cues, but also a general motivation to protect children from harm. Such protection-oriented judgments may be associated with more cautious purchase decisions and lower purchase intention. Evidence from adjacent research helps situate this finding. Eibach and Mock (2011) demonstrated that making the parental role salient increased risk perception and risk-averse choice, while Russell et al. (2015) identified health and nutrition as important considerations when parents selected foods for their children. It also accords with meta-analytic evidence linking food-safety risk perception to consumer choice and willingness to buy (Nardi et al., 2020) and with previous research documenting a negative relationship between perceived risk and purchase intention toward pre-prepared foods (He et al., 2026; Xiong et al., 2023). Building on these findings, the present study connects a protection-oriented parenting motive with consumers’ product risk evaluations and purchase intentions.

Second, the moderated mediation results showed that the negative indirect relationship between parental protection motivation and purchase intention through perceived risk was stronger at higher levels of health consciousness. The mediating effect of perceived risk was significant at high but not low levels of health consciousness. Consumers with greater health consciousness tend to pay closer attention to the potential health consequences of food and apply more stringent health and nutritional standards when evaluating food products. When this health-oriented evaluation coincides with stronger parental protection motivation, concerns about ingredients, additives, nutritional quality, and industrial processing may become more salient, resulting in a stronger association between parental protection motivation and perceived risk. Research on food-related health evaluations supports this interpretation. Siegrist et al. (2022) found that diet-related health consciousness was positively associated with perceived lifestyle risks in the food domain. Devi et al. (2023) further showed that health consciousness strengthened the relationships between risk-related evaluations and organic food purchase intention. The present study develops this line of research by showing that health consciousness also conditions the indirect relationship between a protection-oriented motive and reluctance to purchase pre-prepared foods.

Third, information sensitivity exhibited a contrasting moderated mediation pattern. The negative indirect effect of parental protection motivation on purchase intention through perceived risk was weaker at higher levels of information sensitivity and was significant at low but not high levels of information sensitivity. Consumers with greater information sensitivity place more importance on product-specific evidence, such as ingredient lists, processing methods, and origin information. Consequently, they may evaluate whether protection-related concerns are supported by available product information rather than relying primarily on a general sense of uncertainty. This result can be understood in light of research on food information and uncertainty reduction. Chen and Huang (2013) found that food traceability systems reduced perceived uncertainty and increased purchase intention toward fast foods. Wu et al. (2021) similarly showed that the perceived quality of traceability information was associated with lower uncertainty and greater organic food purchase intention, while the importance consumers attached to product information altered the relationship between uncertainty and purchase intention. Whereas these studies emphasize the availability and quality of food information, the present findings indicate that consumers’ orientation toward such information may also shape the risk-evaluation process. Information sensitivity does not necessarily lower perceived risk directly; rather, it appears to weaken the extent to which general parental protection motivation corresponds to heightened risk perceptions of pre-prepared foods.

## 7 Conclusion

This study examined the relationship between parental protection motivation and consumers’ purchase intention toward pre-prepared foods, focusing on the mediating role of perceived risk and the moderated mediating roles of health consciousness and information sensitivity. Parental protection motivation was positively associated with perceived risk, which was in turn negatively associated with purchase intention. Separate moderated mediation analyses further showed that this indirect relationship was stronger at higher levels of health consciousness but weaker at higher levels of information sensitivity. These findings deepen the protection-oriented explanation of parenting-related motives in consumer behavior and extend research on pre-prepared food consumption by complementing proximate explanations with an ultimate-level motivational antecedent. The study also provides practical implications for pre-prepared food companies in designing differentiated risk communication and trust-building strategies for distinct consumer groups.

### 7.1 Theoretical contributions

First, this study introduces parental protection motivation into research on pre-prepared food consumption as a protection-specific and evolutionarily grounded motivational factor. Existing studies have primarily explained consumers’ responses to pre-prepared foods through proximate product attributes and cognitive evaluations (Liu et al., 2025; Wong et al., 2025; Xiong et al., 2023; Xu and Sun, 2026), whereas consumer research on parenting-related motives has mainly examined broader caregiving, nurturing, and investment tendencies (Chen et al., 2024; Li et al., 2019; Roy et al., 2026). By focusing on the protection component of parental caregiving, the present study connects these two research streams and shows that parental protection motivation is associated with consumers’ risk evaluations and purchase intentions in a context where convenience coexists with uncertainty about food safety and nutritional quality. This protection-oriented account complements, rather than replaces, existing explanations based on product characteristics and immediate consumer evaluations.

Second, this study integrates the evolutionary needs perspective and protection motivation theory by identifying perceived risk as the cognitive mechanism connecting an evolutionarily grounded motive with product-specific purchase intention. The evolutionary needs perspective explains the origin and adaptive function of parental protection motivation, whereas protection motivation theory explains how protection-related concerns are reflected in threat evaluation and behavioral intention (Confer et al., 2010; Rogers, 1975). The mediating role of perceived risk links these two theoretical levels by showing how an ultimate-level motive is associated with purchase intention through a proximate evaluation of a specific food category. This integration clarifies the complementary roles of the two theories rather than treating them as parallel theoretical foundations.

Third, this study identifies differentiated boundary conditions in the indirect relationship between parental protection motivation and purchase intention. Prior research has shown that health consciousness can shape how risk-related evaluations are translated into food purchase intentions (Devi et al., 2023), while the importance consumers attach to product information can alter their responses to product uncertainty (Wu et al., 2021). Building on these studies, the present study examines health consciousness and information sensitivity separately as two boundary conditions of the mediated relationship and reveals their contrasting roles. These findings suggest that consumers’ acceptance or rejection of pre-prepared foods depends not only on their underlying protective motives and risk evaluations, but also on how strongly they prioritize health-related concerns and rely on product information when making purchase decisions. The findings therefore provide a more conditional account of how evolutionarily grounded motives are associated with consumer responses in contemporary food-consumption contexts.

### 7.2 Practical implications

First, companies should develop trust-building strategies grounded in evolutionary psychology to reduce the perceived risks associated with parental protection motivation. Because parental protection motivation is associated with greater perceived risk, firms should present pre-prepared foods not merely as convenient products but as safe, controllable, and reliable options for family meals. This positioning should be supported by standardized production, rigorous quality control, transparent ingredient sourcing, clear disclosure of nutritional composition and additive use, product traceability, and credible third-party certification. Marketing communications should translate these safeguards into specific and verifiable information about how potential food safety and nutritional risks are controlled throughout production and distribution. Such an approach may reduce uncertainty surrounding pre-prepared foods and alleviate consumers’ risk concerns.

Second, the contrasting moderating patterns of health consciousness and information sensitivity highlight the value of considering these characteristics in conjunction when developing product and communication strategies. For consumers with greater health consciousness, product development could emphasize balanced nutritional composition, reduced additive use, and formulations suitable for family or child-related consumption. The presentation of these attributes should also reflect consumers’ information sensitivity. Firms could assess consumers’ information preferences through brief voluntary questions or privacy-respecting indicators, such as whether they access detailed product pages or scan QR codes for additional information. Consumers with high levels of both health consciousness and information sensitivity may prefer detailed and evidence-based information supporting nutritional and safety claims, whereas highly health-conscious consumers with lower information sensitivity may respond more favorably to concise nutritional summaries, recognized certifications, and clearly organized visual labels. This joint approach allows firms to coordinate health-oriented product strategies with the depth and presentation of marketing information.

### 7.3 Limitations and future research

First, this study employed a cross-sectional survey design, which does not establish the temporal ordering or causal direction of the relationships among parental protection motivation, perceived risk, and purchase intention. Accordingly, the mediation and moderated mediation results should be interpreted as relational patterns consistent with the proposed theoretical framework. In addition, the self-report measure assesses current individual differences in parental protection motivation, whose evolutionary grounding is informed by prior theory and research rather than directly tested in the present study. Such measures remain susceptible to subjective bias and potential common-method bias. Future research could use longitudinal designs, adopt scenario-based experiments, or incorporate objective behavioral data, such as actual purchase records or household dietary logs, to improve methodological robustness and provide stronger evidence concerning the proposed relationships.

Second, the composition and cultural scope of the sample may limit the generalizability of the findings. All participants were Chinese consumers, and women accounted for 64.5% of the sample. Although gender was included as a covariate in the statistical analyses, prior research suggests that gender and parenthood may be associated with consumption decisions and food-parenting practices (Davison et al., 2020; Migheli, 2021). Future research could recruit more gender-balanced samples and conduct studies in other cultural contexts to examine gender differences and assess the cross-cultural applicability of the proposed relationships.

Third, this study did not examine other consumer- and information-related factors that may provide alternative explanations. Prior food-consumption research has linked cognitive fluency to perceived risk and purchase intention (Li et al., 2022), traceability knowledge and information quality to perceived risk and food purchase intention (Ge, 2022), and consumer trust to purchase intention in risk-sensitive food contexts (Huo and Liu, 2022). Future research could incorporate these factors to assess the incremental explanatory role of parental protection motivation.

Fourth, pre-prepared foods encompass diverse categories, including ready-to-eat, ready-to-heat, ready-to-cook, and ready-to-serve products (He et al., 2026). These categories vary substantially in freshness, processing level, additive use, brand reputation, and price, which may influence consumers’ risk perceptions differently. Treating pre-prepared foods as a single category may therefore obscure important differences. Future research should distinguish among product types and examine how specific product characteristics moderate the influence of parental protection motivation on perceived risk and purchase intention.

Finally, this study focused primarily on individual-level moderators. Although health consciousness and information sensitivity significantly moderated the relationship between parental protection motivation and perceived risk, pre-prepared food consumption is also influenced by broader social and contextual factors. Future research should incorporate contextual variables, such as food certification labels, brand origin, or regulatory endorsement, to further examine how external cues mitigate perceived risk and influence purchase decisions.

## Data Availability

The raw data supporting the conclusions of this article will be made available by the authors, without undue reservation.

## Footnotes

1 Sina Weibo (http://www.weibo.com) is a major Chinese microblogging and social media platform that supports real-time public posting, reposting, commenting, and topic-based discussion. It is widely used to circulate and discuss news, social events, and consumer issues.
