# Supplementary material for: From protection to rejection: the role of parental protection motivation in pre-prepared food consumption
Source: Front Psychol. 2026 Oct 14;17:1928184. doi: 10.3389/fpsyg.2026.1928184 (PMC13713418; doi:10.3389/fpsyg.2026.1928184)
Supplement: Supplementary file 1 [file Supplementary_file_1.pdf]

Appendix Table A. Overview of prior related studies

Studies	Parenting-related motivation		Antecedents of food consumption		Moderators	
	General parenting/parental care motivation	Parental protection motivation	Proximate factors	Ultimate (evolutionary needs)	Health consciousness	Information sensitivity
**Stream I: Parenting-related motivation and consumer behavior (representatives)**						
Li et al. (2019)	√					
Li and Yan (2021)	√					
Liang et al. (2023)	√					
Caso et al. (2024)	√		√			
Chen et al. (2024)	√					
Roy et al. (2026)	√					
**Stream II: Pre-prepared food consumption (representatives)**						
Xiong et al. (2023)			√			
Liu et al. (2025)			√			
Wong et al. (2025)			√			
He et al. (2026)			√			
Qi et al. (2026)			√			
Xu and Sun (2026)			√			
**This study**		✓		✓	✓	✓

Notes: Studies in each cell are presented in chronological order.

Source: Authors' own work

References

Li, Y. J., Haws, K. L., and Griskevicius, V. (2019). Parenting motivation and consumer decision-making. *J. Consum. Res.* 45, 1117–1137. doi: 10.1093/jcr/ucy038

Li, Y., and Yan, D. (2021). Cuteness inspires men's risk seeking but women's risk aversion. *J. Bus. Res.* 126, 239–249. doi: 10.1016/j.jbusres.2020.12.066

Liang, Y. (Sky), Huang, Z. (Tak), and Su, L. (2023). Too time-crunched to seek variety: the

influence of parenting motivation on consumer variety seeking. *J. Mark. Res.* 60, 812–833. doi: 10.1177/00222437221136491

Caso, G., Annunziata, A., and Vecchio, R. (2024). Indulgent, practical or balanced? Exploring mothers' food choices for their children in low- and middle-income households. *Food Qual. Prefer.* 113:105080. doi: 10.1016/j.foodqual.2023.105080

Chen, Q., Wang, Y., and Zhang, Y. (2024). Developing strengths or remedying weaknesses? How perceived social mobility affects parents' purchase preferences for children's educational products. *J. Mark.* 88, 46–62. doi: 10.1177/00222429231224333

Roy, A., Gera, N., and Das, G. (2026). Safety first! Wait, ambition first! How parental psychology shapes purchase preferences for their children. *Psychol. Mark.* 43, 262–273. doi: 10.1002/mar.70055

Xiong, Y., Lin, X., Wen, X., Wang, Y., Liang, W., and Xing, T. (2023). The analysis of residents' intention to consume pre-made dishes in China: a grounded theory. *Foods* 12:3798. doi: 10.3390/foods12203798

Liu, X., Wang, J., and Liu, C. (2025). Are you still willing to use pre-made dishes? Examining young customers' continuous usage intention in the Chinese emerging market. *Br. Food J.* 127, 73–91. doi: 10.1108/BFJ-07-2024-0693

Wong, J. W. C., Song, Y., Xu, L., and Lu, A. Y. E. (2025). How do prepared dishes attract customers? Effects of prepared dish attributes on customers' trust, attitudes and adoption intentions. *Br. Food J.* 127, 4606–4625. doi: 10.1108/BFJ-03-2025-0290

He, Q., Zhao, Y., Sun, Y., and Ji, H. (2026). Consumer perceptions of pre-prepared food safety risks: evidence from survey experiments. *J. Sci. Food Agric.* 106, 1744–1756. doi: 10.1002/jsfa.70307

Qi, X., Zhao, H., and Yu, X. (2026). Predicting consumer purchase intention for pre-prepared meals based on random forest and explainable AI (SHAP): a study in Jilin Province, China. *Foods* 15:896. doi: 10.3390/foods15050896

Xu, Z., and Sun, S. (2026). The influence of front-of-package label types on consumer purchase intentions for prepared dishes. *Br. Food J.* 128, 3096–3114. doi: 10.1108/BFJ-01-2026-0016

Appendix Table B. Measurement items

Purchase intention toward pre-prepared foods (Dodds *et al*., 1991)

1. I will consider purchasing pre-prepared food.
2. My willingness to buy pre-prepared food is very high.
3. The likelihood of purchasing pre-prepared food is very high.

Parental protection motivation (Buckels *et al*., 2015)

1. I would hurt anyone who was a threat to a child.
2. I would show no mercy to someone who was a danger to a child.
3. I would use any means necessary to protect a child, even if I had to hurt others.
4. I would feel compelled to punish anyone who tried to harm a child.
5. I would sooner go to bed hungry than let a child go without food.

Perceived risk (Stone and Grønhaug, 1993)

1. Overall, the thought of buying pre-prepared food causes me to be concerned with experiencing some kind of loss.
2. All things considered, I think I would be making a mistake if I bought pre-prepared food.
3. When all is said and done, I really feel that the purchase of pre-prepared food poses problems for me that I just don't need.

Health consciousness (Michaelidou and Hassan, 2008)

1. I reflect about my health a lot.
2. I'm very self-conscious about my health.
3. I'm alert to changes in my health.
4. I take responsibility for the state of my health.

Information sensitivity (Bansal *et al*., 2010)

1. I am sensitive to the information about food ingredient list.
2. I am sensitive to the information such as the production date or shelf life.
3. I am sensitive to the information about food origin safety.
4. I am sensitive to the information about food processing methods.
5. I am sensitive to the information on food nutrition labels.

Appendix Table C. Heterotrait–monotrait ratio of correlations (HTMT)

	1	2	3	4
1. Purchase intention				
2. Parental protection motivation	0.187			
3. Perceived risk	0.694	0.304		
4. Health consciousness	0.318	0.291	0.224	
5. Information sensitivity	0.385	0.459	0.395	0.412
